# Vision and Hearing Impairment in Older Adults and Outdoor Mobility Limitations: The Role of Public Transit

**DOI:** 10.1093/geroni/igaf122.078

**Published:** 2025-12-31

**Authors:** Philippa Clarke, Shu Xu, Nasya Tan, Joshua Ehrlich

**Affiliations:** University of Michigan, Ann Arbor, Michigan, United States; University of Michigan, Ann Arbor, Michigan, United States; University of Michigan, Ann Arbor, Michigan, United States; University of Michigan, Ann Arbor, Michigan, United States

## Abstract

Sensory loss in older adults poses a risk for mobility limitations. Yet, little research has examined how the accessibility of the surrounding built environment, as measured through access to public transportation, mitigates the risk of mobility limitations for those with vision impairment, hearing impairment, or dual sensory impairment (both vision and hearing impairment). In this paper we examined the frequency of leaving one’s home among older adults (age 65+) with objectively measured vision and hearing impairment (and dual impairment) in Round 12 (2022) of the National Health and Aging Trends Study (n = 5185), and examined the modifying role of the density of public transit stops in the local neighborhood. Weighted logistic regression models (adjusted for demographics, living arrangements, and health conditions) found that older adults with hearing impairment had 30% lower odds (OR = 0.70, 95% CI = 0.52-0.93) of leaving their home (every/most days in the past week vs. never/rarely) compared to older adults with no sensory impairment. Those with both vision and hearing impairment had a 53% lower odds of leaving their home (OR = 0.47, 95% CI = 0.32-0.69). However, these associations were attenuated when older adults with hearing impairment or dual sensory impairment lived in a neighborhood with at least one public transit stop. Findings emphasize the importance of considering dual sensory impairment in older adults and the potential role of the surrounding environment for mitigating the risk of mobility restrictions with aging.

